# Effect of radiochemotherapy on T2* MRI in HNSCC and its relation to FMISO PET derived hypoxia and FDG PET

**DOI:** 10.1186/s13014-018-1103-1

**Published:** 2018-08-29

**Authors:** Nicole Wiedenmann, Hatice Bunea, Hans C. Rischke, Andrei Bunea, Liette Majerus, Lars Bielak, Alexey Protopopov, Ute Ludwig, Martin Büchert, Christian Stoykow, Nils H. Nicolay, Wolfgang A. Weber, Michael Mix, Philipp T. Meyer, Jürgen Hennig, Michael Bock, Anca L. Grosu

**Affiliations:** 1 Department of Radiation Oncology, Medical Center University of Freiburg, Faculty of Medicine, University of Freiburg, Freiburg, Germany; 2 Department of Radiology, Medical Physics, Medical Center University of Freiburg, Faculty of Medicine, University of Freiburg, Freiburg, Germany; 3 Department of Nuclear Medicine, Medical Center University of Freiburg, Faculty of Medicine, University of Freiburg, Freiburg, Germany; 4 German Cancer Consortium (DKTK), Partner Site Freiburg, Freiburg, Germany; 5 German Cancer Research Center (DKFZ), Heidelberg, Germany; 6 Clinic for Nuclear Medicine, Technische Universität München, Munich, Germany

**Keywords:** Tumour hypoxia, T2*, Multiparametric MRI, FMISO PET, FDG PET, HNSCC

## Abstract

**Background:**

To assess the effect of radiochemotherapy (RCT) on proposed tumour hypoxia marker transverse relaxation time (T2*) and to analyse the relation between T2* and ^18^F-misonidazole PET/CT (FMISO-PET) and ^18^F-fluorodeoxyglucose PET/CT (FDG-PET).

**Methods:**

Ten patients undergoing definitive RCT for squamous cell head-and-neck cancer (HNSCC) received repeat FMISO- and 3 Tesla T2*-weighted MRI at weeks 0, 2 and 5 during treatment and FDG-PET at baseline. Gross tumour volumes (GTV) of tumour (T), lymph nodes (LN) and hypoxic subvolumes (HSV, based on FMISO-PET) and complementary non-hypoxic subvolumes (nonHSV) were generated. Mean values for T2* and SUVmean FDG were determined.

**Results:**

During RCT, marked reduction of tumour hypoxia on FMISO-PET was observed (T, LN), while mean T2* did not change significantly. At baseline, mean T2* values within HSV-T (15 ± 5 ms) were smaller compared to nonHSV-T (18 ± 3 ms; *p* = 0.051), whereas FDG SUVmean (12 ± 6) was significantly higher for HSV-T (12 ± 6) than for nonHSV-T (6 ± 3; *p* = 0.026) and higher for HSV-LN (10 ± 4) than for nonHSV-LN (5 ± 2; *p* ≤ 0.011). Correlation between FMISO PET and FDG PET was higher than between FMSIO PET and T2* (R^2^ for GTV-T (FMISO/FDG) = 0.81, R^2^ for GTV-T (FMISO/T2*) = 0.32).

**Conclusions:**

Marked reduction of tumour hypoxia between week 0, 2 and 5 found on FMISO PET was not accompanied by a significant T2*change within GTVs over time. These results suggest a relation between tumour oxygenation status and T2* at baseline, but no simple correlation over time. Therefore, caution is warranted when using T2* as a substitute for FMISO-PET to monitor tumour hypoxia during RCT in HNSCC patients.

**Trial registration:**

*DRKS,* *DRKS00003830* *. Registered 23.04.2012.*

**Electronic supplementary material:**

The online version of this article (10.1186/s13014-018-1103-1) contains supplementary material, which is available to authorized users.

## Background

In squamous cell carcinoma of the head and neck (HNSCC) assessment of the extent of tumour hypoxia under primary radiochemotherapy (RCT) is warranted to obtain an early prognostic marker and to define potential dose escalation volumes [1–14]. Positron emission tomography (PET) can be considered the gold standard method for hypoxia imaging using hypoxia-associated tracers such as [^18^F]-fluoromisonidazole (FMISO) and [^18^F]-fluoroazomycinarabinoside (FAZA) [15–18]. Magnetic resonance imaging (MRI) can be used to characterize tumour function in several ways: Gadolinium(Gd)-perfusion MRI analyzes the tumour perfusion using the dynamic signal change after contrast medium injection, while Blood Oxygen Level Dependent (BOLD) MRI aims at assessing oxygen consumption. The MRI apparent transverse relaxation time T2*, respectively its reciprocal the relaxation rate R2*, obtained from T2*-weighted MRI, have been proposed as a potential imaging biomarker and surrogate for hypoxia PET [19–34]. A change in regional concentration of oxy- vs. deoxyhaemoglobin can result in a change in magnetic field homogeneity, which is leading to a signal change in T2*-weighted MR acquisitions.

In the literature, controversial findings are reported for T2* at baseline and during RCT: T2*-weighted MRI has recently been compared to FMISO-PET in glioma patients [32] where it provided complementary information rather than spatial correlation. In cervix cancer patients Kim et al. studied BOLD MRI before and after RCT, and they report a correlation between tumour R2* pre-RCT with tumour size response but not with tumour volume response [29]. Li et al. identified tumour R2* before RCT as a significant prognostic factor for progression-free and overall survival [30]. In HNSCC patients undergoing RCT, Panek et al. examined T2* signal stability and reproducibility pre-RCT, and they found that T2* measurements are highly reproducible [23]. Min et al. and Wong et al. evaluated serial functional imaging including R2*/T2*-weighted MRI in HNSCC during RCT and found no clear pattern for changes in R2* [33, 34].

Here, we assessed hypoxia by analyzing T2* as a measure of the deoxyhaemoglobin concentration and used T2-weighted sequences and Gd-contrast enhanced T1 sequences for morphological characterization and delineation of tumours and lymph node metastasis. The aim of our study was to examine the effect of RCT on T2* in HNSCC at an early and late time point during RCT and to analyse the relation between T2* and FMISO-PET. Serial imaging was scheduled before RCT and at week 2 and week 5 during RCT. Baseline ^18^F-fluorodeoxyglucose-PET/CT (FDG-PET) was included to optimize pretherapy staging and considered for image analysis. To our knowledge, this is the first study to combine T2*-weighted MRI with FMISO-PET in HNSCC.

## Methods

### Patients, imaging schedule and treatment

Thirty two patients (T2–4 N+) were enrolled for this prospective functional MRI and hypoxia PET/CT imaging study during definitive RCT for HNSCC. Patients were recruited from 08/2014 to 11/2015. RCT was administered for 7 weeks in daily fractions of 2 Gy to a total dose of 70 Gy to the primary tumour and macroscopic lymph node metastases and 50 Gy to the elective lymphatic drainage. Concurrent chemotherapy was administered once in weeks 1, 4, and 7 with cisplatin (100 mg/kg/d or adjusted to lower dose) or carboplatin in case of chronic renal insufficiency.

Patients underwent serial FMISO-PET as previously described [7, 8] and MRI in weeks 0, 2 and 5. FDG-PET was conducted in week 0. From the total patient cohort, 10 patients (Additional file 1: Table S1) met inclusion criteria for functional MRI image analysis, i.e. imaging with repeat 3 Tesla MRI and presence of a complete set of serial FMISO-PET data.

### PET/CT imaging

[^18^F]-FMISO and [^18^F]-FDG production met standard quality criteria (tracer synthesis: Euro-PET GmbH, Freiburg, Germany). All patients received an injection of 243–332 MBq (6.6–9.0 mCi) ^18^F-MISO and of 303–471 MBq (8.2–12.7 mCi) ^18^F-FDG, respectively. In case of ^18^F-FDG whole body PET/CT scans were performed 1 h p.i. (2 min per bed position, 288 × 288 matrix) with contrast-enhanced diagnostic CT (120 keV, 100–250 mAs, dose modulation, 600 mm data collection diameter, 512 × 512 matrix) and 2 mm slice thickness from the base of the skull to the proximal femur (Gemini TF Big Bore, Philips Healthcare, Cleveland, USA). A subsequent low-dose PET/CT scan was performed in one bed position (10 min) covering the head and neck region. Static ^18^F-MISO PET/CT was executed in one bed position (20 min) covering the head and neck region 160 min p.i., following our previous study [7]. PET data were corrected for scatter, attenuation, randoms and decay and expressed as standardized uptake value (SUV; i.e. local radioactivity concentration normalized to injected dose per body weight). Head and neck FDG SUV images were normalized to mean uptake in normal tissue, defined as spherical volume within the contralateral sternocleidomastoid muscle.

### MRI imaging and calculation of T2* parameter maps

As part of a multi-parameter MR imaging protocol consisting of T1w-MRI, T2w-MRI, dynamic contrast enhanced (DCE-MRI) perfusion measurements and diffusion weighted measurements (DWI-MRI), and multi-echo fast spoiled gradient echo (FLASH) data were acquired on a clinical 3 Tesla MRI system (Tim Trio, Siemens). For this, patients were positioned on the MR system table in the MR-compatible immobilization mask, an anterior 4-element flexible radiofrequency receiver coil (Flex Loop Large) was wrapped around the head-and-neck region, and data were acquired in combination with the posterior spine coil elements within the patient table to maximize the local signal-to-noise ratio at the tumour. The multi-echo acquisition for T2* measurements was acquired prior to the DCE measurements, and imaging slices were defined in transverse orientation. The following imaging parameters were used: 22 slices, spatial resolution: 3 (slice thickness) × 1.1 × 1.1 mm^3^, matrix size: 256^2^, 12 echoes, monopolar readout gradients, echo times TE = 4.83–33.0 ms, echo spacing ΔTE = 2.55 ms, repetition time TR = 400 ms, flip angle α = 60°, readout bandwidth = 815 Hz/pixel, total acquisition time = 1:42 min:s.

From the multi-echo FLASH image series, a T2* map was calculated for each slice position by a pixel-wise mono-exponential (S(TE) = S_0_ exp.(−TE/T2*)) fit to the signal. To avoid noise bias, signal intensities in later echoes that were smaller than 5 times the noise level were not used for fitting.

For all image acquisitions (FDG-PET, FMISO-PET, MRI) patients were immobilized identical to the radiation position with individually casted head and neck masks.

### Contouring and image analysis

Image data were transferred to iplan net planning software (BrainLAB) and co-registered. The GTV for primary tumour (GTV-T) and pathological lymph nodes (GTV-LN) for weeks 0, 2, and 5 were contoured within MRI images based on contrast-enhanced T1w-MRI Gd (Gadolinium) and T2 sequences as consensus volumes between a board certified radiologist and radiation oncologist. At week 0, the FDG-PET additional information was considered for GTV contouring. A representative normal tissue volume (NT) was contoured as a sphere within the contralateral sternocleidomastoid muscle. Hypoxic tumour subvolumes (HSV-T), hypoxic lymph node subvolumes (HSV-LN), and complementary non-hypoxic tumour subvolumes and non-hypoxic lymph node subvolumes (nonHSV-T, nonHSV-LN) were generated for a threshold level of 1.4 times the FMISO SUVmean within the NT. Volumes were contoured for all time points (week 0, 2 and 5) individually. Average values and standard deviation for T2* values and FDG SUVmean were obtained within the volumes NT, GTV-T, GTV-LN, and hypoxic subvolumes HSV-T, HSV-LN and complementary non-hypoxic subvolumes nonHSV-T and nonHSV-LN, respectively (Additional file 2: Figure S1).For statistical analysis IBM SPSS Statistics version 24.0.0.1 and SigmaPlot Version 8.02 were used. A paired samples t-test was applied to compare mean values and test for statistical significance (95% confidence interval). For correlation analysis, R^2^ was calculated using linear regression analysis (SigmaPlot). For week 0, correlations between FMISO uptake, FDG uptake and T2* were analysed by correlating SUVmax tumour/SUVmean muscle for FMISO and FDG with each other and with mean T2* signal: within GTV-T (respectively within GTV-LN), R^2^ (FMISO to T2*) was calculated for FMISO SUVmax GTV-T/SUVmean NT to T2* mean GTV-T and R^2^ (FMISO to FDG) was calculated for FMISO SUVmax GTV-T/SUVmean NT to FDG SUVmax GTV-T/SUVmean NT. For weeks 2 and 5, correlations between FMISO uptake and T2* were analysed.

### Legal issues

The study was approved by the Federal Office for Radiation Protection, the Federal Institute for Drugs and Medical Devices, and the local ethical committee. Production and use of PET radiopharmaceutical [^18^F]-FMISO was approved and registered at the appropriate authority.

## Results

### GTV and HSV under RCT

**Fig. 1:**
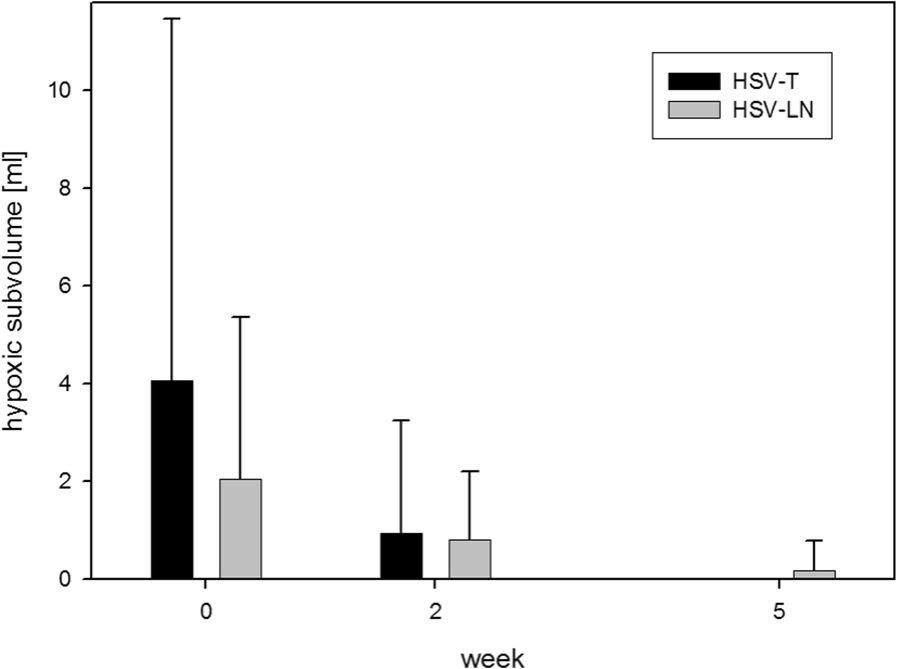
*Hypoxic tumour- and lymph node subvolumes.* Marked reduction of tumour hypoxia during RCT for both HSV-T and HSV-LN between week 0 to week 5

**Fig. 2:**
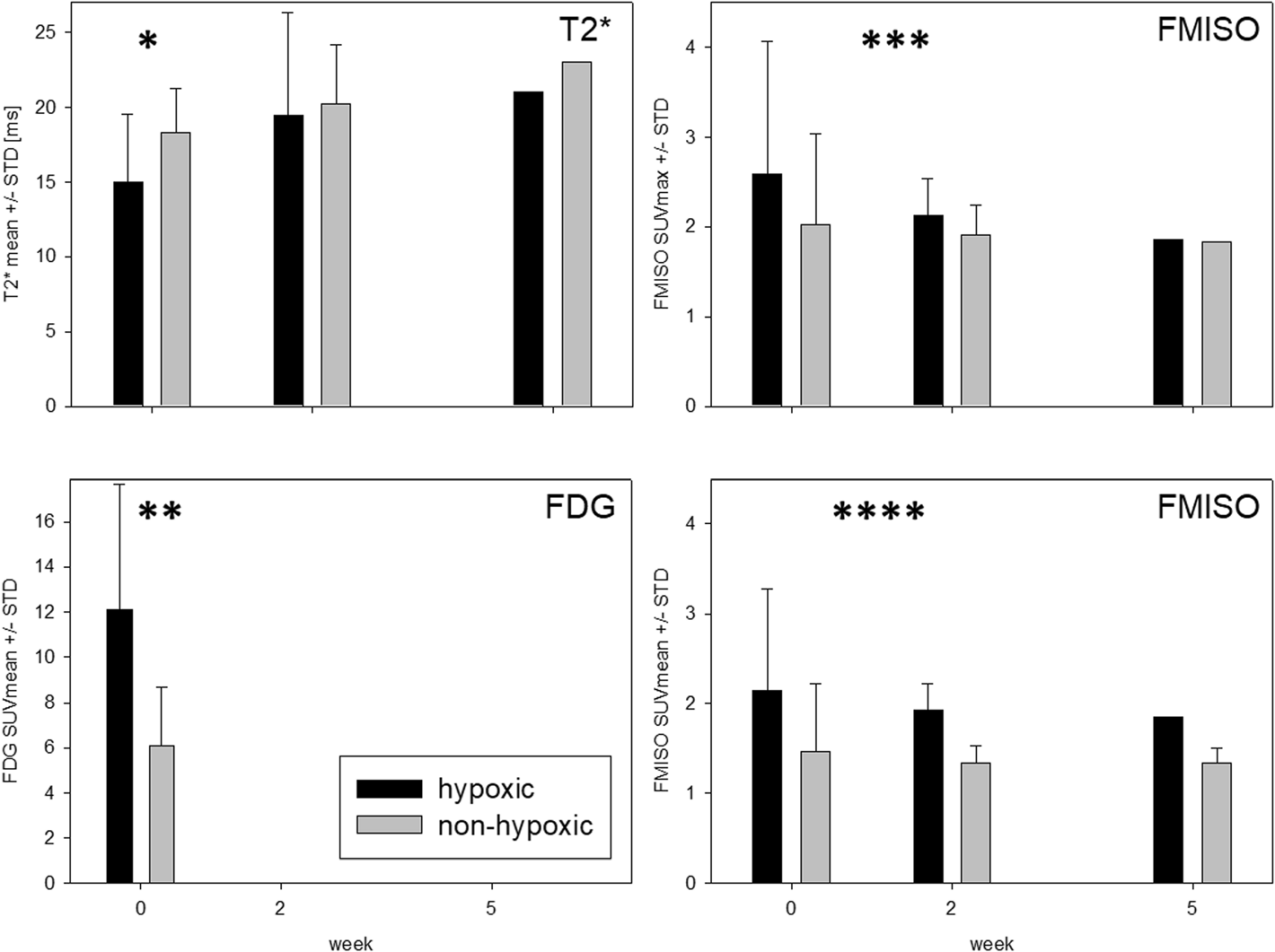
Representative example of imaging modalities MRI T1, T2*, and FMISO-PET. Primary tumour and lymph node metastasis (pt. 5, tonsillar carcinoma) at week 0, 2, and 5 (upper, middle, lower panel): co-registered image sets from MRI T1, MRI T2*, FMISO-PET (left to right). Red contours: GTV-T, GTV-LN. Blue contour: HSV-LN

10/32 patients met inclusion criteria for image analysis by presenting a complete set of serial FMISO-PET and at least two serial 3 Tesla MRI image sets. Images were co-registered and volumes for GTV-T, GTV-LN, and NT were delineated within T1w-MRI Gd, with T2 and FDG-PET being co-registered. The number of lymph nodes present in the GTV-LN is shown in Additional file 3: Table S2. HSV were obtained within FMISO-PET as described and are shown in Table 1. GTV-T and GTV-LN more than halved from week 0 to 5. Hypoxic subvolumes HSV-T and HSV-LN decreased over time and nearly completely resolved (see Table 1, Fig. 1). A representative example of imaging modalities (T1w-MRI, T2* MRI and FMISO-PET) showing reduction of GTV during RCT and location of HSV is demonstrated in Fig. 2.

**Table 1:** Tumour and lymph node volumes, hypoxic subvolumes

week	GTV-T [ml]	GTV-LN [ml]	HSV-T [ml]	HSV-LN [ml]
0	37 ± 22	16 ± 14	4 ± 7	2.1 ± 3.3
2	24 ± 15	14 ± 14	1 ± 2	0.8 ± 1.4
5	16 ± 11	7 ± 6	0.001 ± 0.004	0.19 ± 0.59
∆ 0–5	- 57%	- 56%	- 100%	- 91%

Mean volumes (± STD) for tumour (GTV-T), lymph nodes (GTV-LN) and hypoxic subvolumes delineated on FMISO-PET (HSV-T, HSV-LN) before (week 0) and during radiochemotherapy (week 2 and 5)

### Mean T2*

**Fig. 3:**
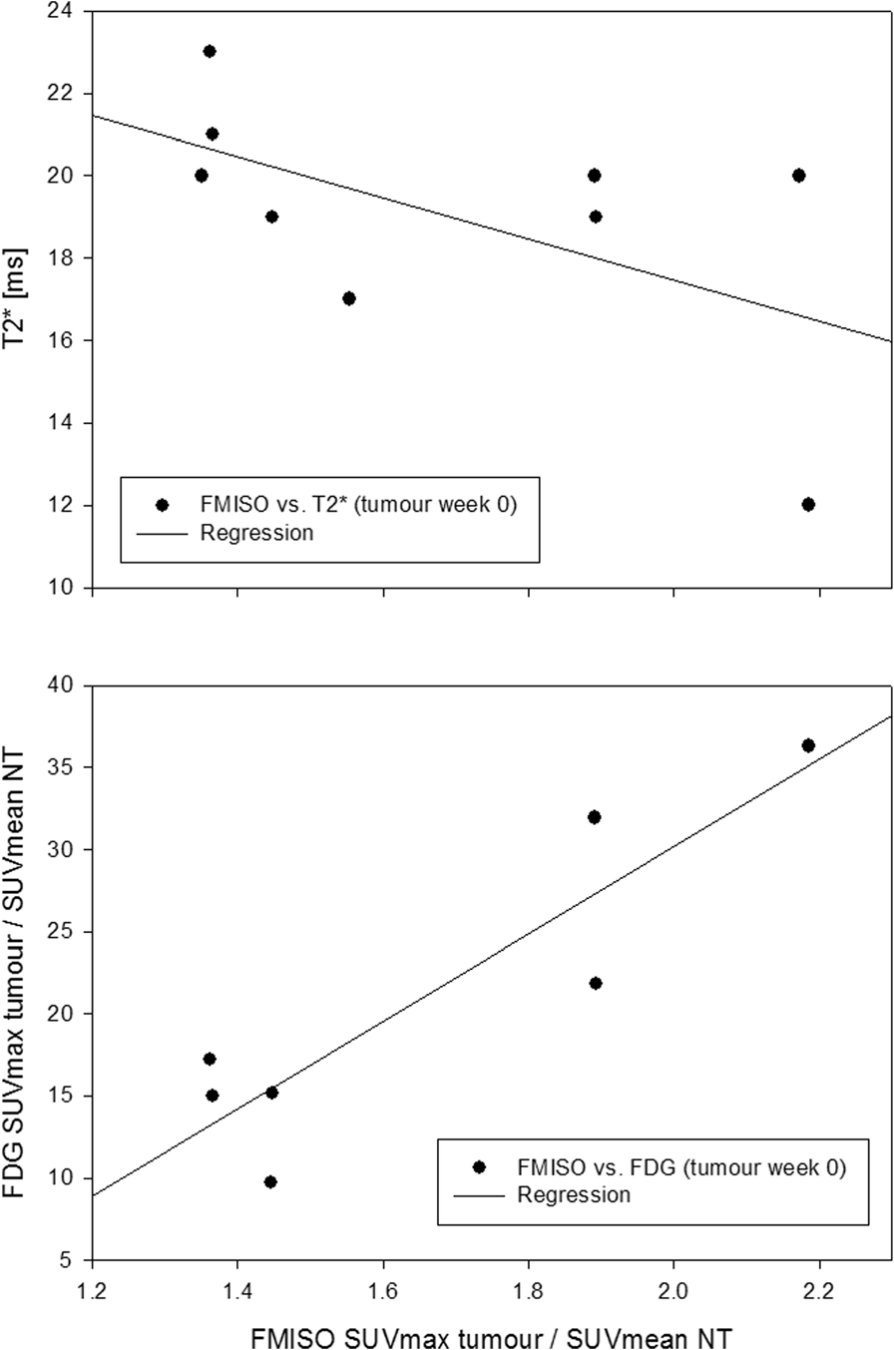
Time course of T2* values within volumes. T2* mean ± STD within tumour, lymph nodes and normal tissue for all patients (*n* = 10)

Mean values for T2* were obtained within designated volumes for each patient (Fig. 3, Additional file 4: Table S3 and Additional file 5: Figure S2). For mean T2* no significant difference was seen between GTV-T and NT for all time points (*p* = 0.239 to 0.879). GTV-LN showed significantly higher mean T2* at week 2 compared to NT and GTV-T: 27.6 ms for GTV-LN compared to 21.0 ms for NT (*p* = 0.021) and 19.1 ms for GTV-T (*p* = 0.009). Mean T2* showed no significant change over time for GTV-T while for GTV-LN a slight decrease week 2 to 5 was seen (*p* = 0.049). GTV and HSV contours in MRI T2* as well as the range of T2* values are visualized in Additional file 6: Figure S3.

### Hypoxic vs. non-hypoxic subvolumes

**Fig. 4:**
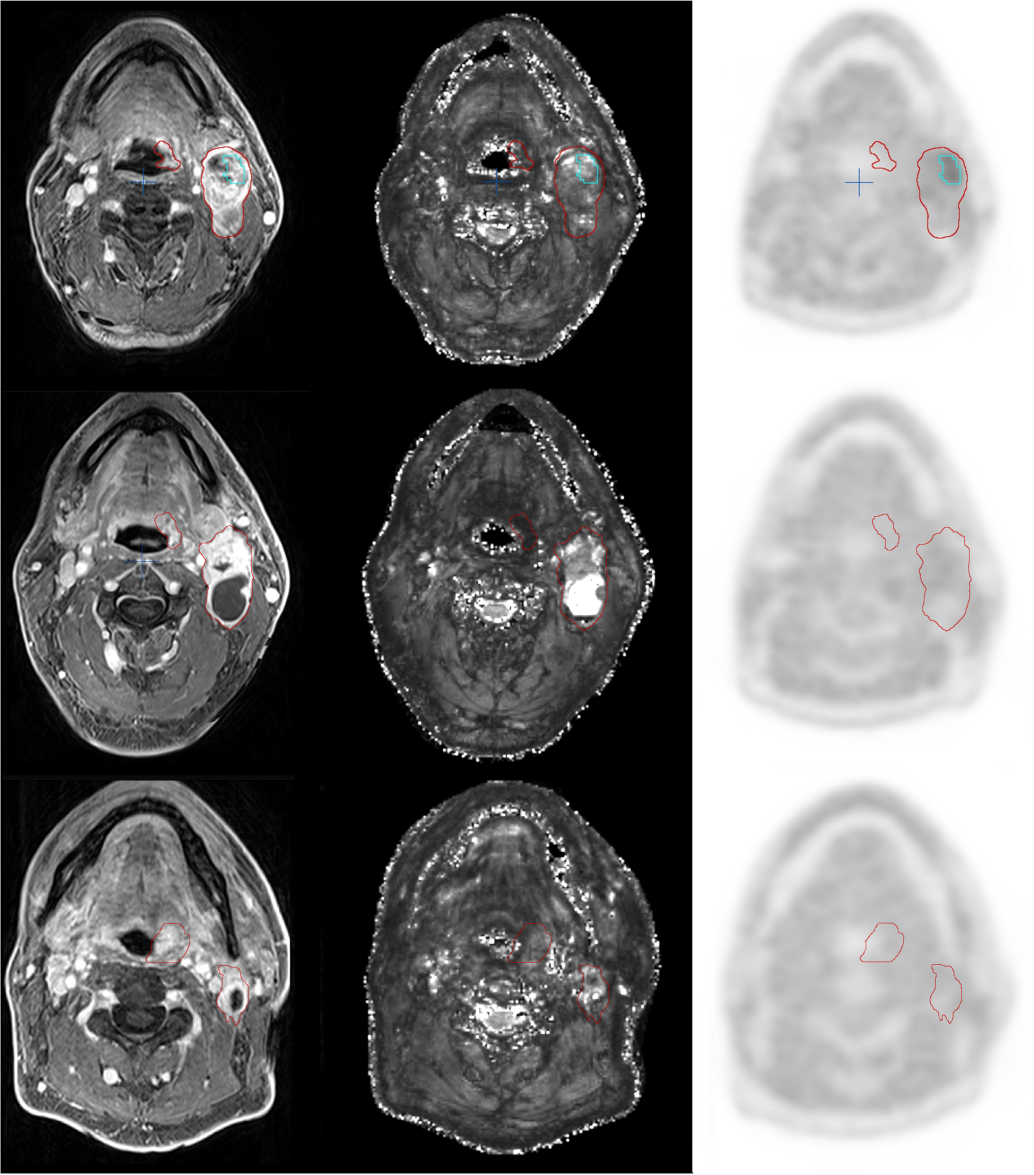
Hypoxic tumour subvolumes: T2* values vs. *FDG uptake and FMISO uptake.* T2* values (ms) were lower and FDG uptake was higher within hypoxic tumour subvolumes as compared to non-hypoxic tumour subvolumes (*p = 0.051, **p = 0.026). FMISO uptake was higher within hypoxic tumour subvolumes than within non-hypoxic tumour subvolumes (***p = 0.029, *p* = 0.072, ****p = 0.003, *p* = 0.0001)

Hypoxic and complementary non-hypoxic tumour and lymph node subvolumes derived from FMISO-PET were compared for T2* signal and FDG SUVmean. HSV-T showed smaller mean T2* values compared to nonHSV-T for all time points, reaching borderline significance level at week 0 (15.0 ± 4.6 ms for HSV-T versus 18.3 ± 2.9 ms for nonHSV-T, *p* = 0.051), see Fig. 4. T2* values within HSV-LN and nonHSV-LN were not significantly different for all time points (*p* = 0.895, *p* = 0.545, Additional file 4: Table S3). FDG SUVmean was significantly higher within hypoxic regions of both tumour and lymph nodes: HSV-T 12.1 ± 5.5 as compared to nonHSV-T 6.1 ± 2.6 and HSV-LN of 10.2 ± 3.9 as compared to nonHSV-LN of 4.7 ± 1.9 (*p* = 0.026 and *p* = 0.011). By definition, FMISO uptake was higher within hypoxic tumour subvolumes as compared to non-hypoxic tumour subvolumes. See Fig. 4 for GTV-T FMISO SUVmean and FMISO SUVmax. Within HSV-T, T2* steadily increased from week 0 to 5, while within HSV-LN a slight decrease was seen. Neither of these changes reached significance level on a paired t-test (*p* = 0.53 and *p* = 0.94).

### Correlation analysis

**Fig. 5:**
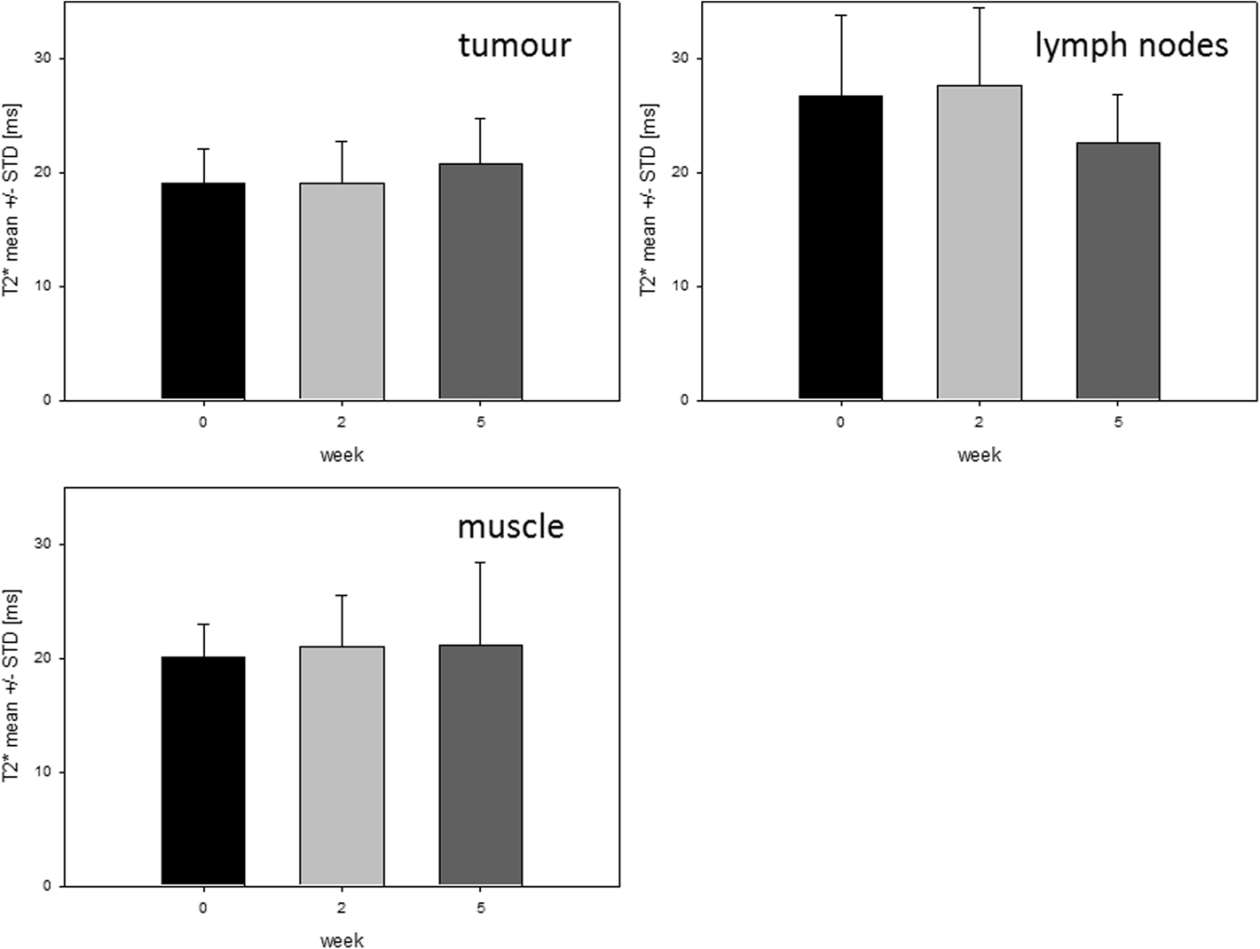
Correlation of FMISO uptake with mean T2* and FDG uptake. Plots showing correlation within GTV-T at baseline

For GTV-T at week 0, the linear regression coefficient R^2^ between FMISO and FDG was 0.81 (*p* = 0.0054), and 0.32 between FMISO and T2* (*p* = 0.1157). For GTV-LN, the comparison between FMISO and FDG yielded an R^2^ of 0.30 (*p* = 0.2057), and an R^2^ of 0.51 (*p* = 0.0459) between FMISO and T2*. At weeks 2 and 5, R^2^ between FMISO and T2* was 0.37 (*p* = 0.1073) and 0.12 (*p* = 0.3663) for GTV-T, and 0.04 (*p* = 0.6334) and 0.01 (*p* = 0.8089) for GTV-LN. Correlation plots for GTV-T at baseline are shown in Fig. 5.

## Discussion

T2* measurements are being considered a novel non-invasive MRI imaging marker for tumour oxygenation [19–34] whereas FMISO-PET is among the most commonly used imaging modalities for non-invasive hypoxia imaging [1–13, 35–38] and could be considered the gold standard. In this prospective longitudinal imaging study we investigated the effect of RCT on T2* values of primary tumours and lymph node metastasis of HNSCC. The imaging protocol included serial MR measurements of T2* and FMISO-PET imaging at 3 time points during definitive RCT. This study is among the first to analyse the effect of radiation on T2* and the first to combine FMISO imaging with T2* measurements in HNSCC.

The more than 50% reduction of GTV-T and GTV-LN up to week 5 and the marked reduction of tumour hypoxia on FMISO-PET (i.e. HSV) in this study are in line with previous findings [4, 7, 8]. Specifically, the pronounced reduction of HSV for both tumour and lymph node metastasis at week 2 and nearly complete resolution at week 5 can be interpreted as tumour reoxygenation. Contrary to the time course of FMISO, we did not see significant changes for T2* over time: A significant difference was only seen for GTV-LN between week 2 and 5, possibly a statistical artefact due to a slight T2* increase from week 0 and 2, as between weeks 0 to 5 there was no significant change. The absence of significant changes for T2* over time is in contrast to the dynamic changes seen with FMISO-PET. While it is possible to stratify patients according to their reoxygenation pattern found on FMISO-PET, this is currently not possible with T2*. For methodological reasons, different endpoints had to be used for FMISO-PET derived hypoxia (hypoxic volumes) and T2* MRI derived oxygenation status (average value within volumes).

It is of note that for GTV-LN higher T2* values were found than for GTV-T for all time points (reaching significance level at week 2). In line with this finding, Panek et al. [23] also described higher T2* values for lymph node metastasis than for primary tumours. Possible explanations might be local magnetic field inhomogeneity caused by air–tissue interfaces that affects T2* values at the outer rim of primary tumours more than that of lymph nodes, as the primary tumours are closer to the air-filled structures of the pharynx and the airways. In addition, T2* is also affected by the T2 value of the tissue which is different for tumour and lymph node.

The comparison between FMISO-PET derived hypoxic tumour subvolumes to complementary non-hypoxic tumour subvolumes showed smaller T2* values for HSV as compared to non-HSV for all time points. This might indicate a correlation between oxygenation status and T2* before therapy. Statistical significance was not reached, however a borderline significance level (*p* = 0.051). Our findings on T2* values within HSV over time were opposed for GTV-T and GTV-LN: within hypoxic tumour volumes, T2* steadily increased from week 0 to 5, supportive for improved tumour oxygenation over time, whereas within hypoxic lymph node subvolumes a slight decrease was seen (none of these changes were significant).

FDG-PET was conducted only once (week 0) and not at later time points for patient convenience. FDG uptake within hypoxic subvolumes was significantly higher than within complementary non-hypoxic subvolumes for both tumour and lymph nodes. Accordingly, within GTV-T the correlation between FMISO uptake and FDG uptake was strong (R^2^ = 0.81) whereas only a weak correlation (R^2^ = 0.32) was found between FMISO uptake and mean T2*. Interestingly, for lymph nodes the correlation between FMISO and T2* was moderate (R^2^ = 0.51) while the correlation between FMISO and FDG was weak (R^2^ = 0.30).

Due to the overlap between FDG avid regions and hypoxic regions on FMISO-PET [8, 39], FDG-PET has been discussed as a potential surrogate for FMISO-PET for the definition of a dose escalation volume. Based on our data of primary tumours, we can support the finding of a correlation between FDG uptake and FMISO uptake. The correlation between T2* and FMISO uptake was less pronounced.

The effective transverse relaxation time, T2*, and its inverse, the transverse relaxation rate, R2* = 1/T2*, are influenced by the presence of paramagnetic deoxyhaemoglobin in venous blood. Thus, deoxyhaemoglobin serves as a naturally occuring contrast agent that allows quantification of oxygenation [20, 22, 25]. Changes in T2* are stronger in large vessels than in microvasculature. For brain imaging at 1.5 Tesla, short T2* down to 10 ms were reported in the sagittal sinus compared with 25–50 ms in larger arterioles and venules [24, 40].

Punwani et al. measured T2* in the brain of neonatal piglets and found a strong correlation between R2* and the absolute deoxyhaemoglobin concentration [25]. Panek et al. investigated T2* at baseline before onset of radiation therapy in ten HNSCC patients and found T2* at 3 Tesla applicable to assess clinically relevant changes in tumour oxygenation [23]. Additionally, T2* measurements were applied in a mouse tumour model and R2* fluctuations were described by the authors in both xenografts and patient tumours [41]. The same group used T2* to assess the effect of blood transfusions on tumour oxygenation in HNSCC patients, revealing no change in tumour oxygenation after transfusion [42]. Spontaneous fluctuations of T2* signal were also reported by Baudelet et al. in a mouse tumour model [19]. This observation was found within regions of functional vasculature and therefore interpreted as spontaneous fluctuations in blood flow and oxygenation associated with the pathophysiology of acute hypoxia in tumours [19]. Li et al. investigated R2* for predicting the prognosis of cervical squamous carcinoma treated with RCT and found significantly lower R2* values for responders compared to the non-responders [30]. Contrarily in HNSCC during RCT, Wong et al. found no significant difference between R2* for responders and non-responders [34] and Min et al. found no clear pattern for changes in R2* by analyzing intra-tumour ROIs [33]. For cervical cancer patients, Kim et al. reported significantly lower R2* values pre-RCT than post-RCT, indicating increased tumour hypoxia after treatment - a finding that was interpreted as caused by increased deoxyhaemoglobin concentration through reduction in vascular permeability and blood flow after treatment [29]. In a preclinical rat tumour model, Hallac et al. was able to correlate R2* obtained by blood oxygen level dependent (BOLD) MRI with pO_2_ [43]. In our study, no distinct correlation between hypoxia as derived from FMISO-PET and T2* was found. As tumour size in HNSCC during RCT is rapidly changing, the change in tumour size and composition might be a possible explanation for this discrepancy. In addition, T2* measurements are strongly affected by the amount of blood supply (perfusion, vascular permeability) and deoxyhaemoglobin concentration, which might change during RCT. Appropriately, in a recent study in prostate cancer patients undergoing neoadjuvant androgen deprivation therapy, assessment of hypoxia by FMISO-PET and MRI-based perfusion showed a correlation at baseline only but not following therapy [44].

There are some limitations to the present study: 1. optimal image fusion is crucial for correlation analysis. MRI and PET/CT images (both with identical mask fixation of the head and neck region) were fused by a treatment planning software with validated image registration algorithm. However, image fusion within the head and neck region is more critical than intracranial image fusion due to anatomical and physiological reasons. 2. The hypoxic subvolumes used for analysis of average T2* values were representative at baseline but became very small at later time points. 3. The study evaluates a relatively small number of patients. Including a higher number of patients however would raise an ethical problem due to the burden imposed on patients by frequent imaging sessions and long imaging protocols. In the current study, 11 of 32 patients could not be considered as they were included before availability of the 3 Tesla MRI. For the remaining 21 patients, a high dropout rate was found, as only 10 fulfilled inclusion criteria due to missing image sets.

Nevertheless, this prospective trial demonstrated that clinically, T2* may not be suitable to replace FMISO PET as a valuable surrogate imaging marker to monitor tumour oxygenation and hypoxic areas during the course of radiotherapy. Future analyses are planned to further examine the role of T2* imaging in the context of tumour hypoxia.

## Conclusions

In summary, marked reduction of tumour hypoxia between week 0, 2 and 5 found on FMISO-PET was not accompanied by a significant T2*change within GTVs over time. Our results suggest a relation between tumour oxygenation status and T2* at baseline, but no simple correlation during the course of radiotherapy. Therefore, caution is warranted when using T2* as a substitute for FMISO-PET to monitor tumour hypoxia during RCT in HNSCC patients.

## Additional files

Additional file 1: **Table S1.** Patient characteristics. (DOCX 15 kb)

Additional file 2: **Figure S1.** *Volumes analysed.* FMISO-PET with volumes used for analysis: GTV-T, GTV-LN, HSV-T, nonHSV-T, HSV-LN, nonHSV-LN. (TIF 216 kb)

Additional file 3: **Table S2.** T2* values within volumes. Mean, median, and STD for T2*mean (ms) measurements for all patients (*n* = 10). (DOCX 15 kb)

Additional file 4: **Table S3.** Number of lymph nodes within GTV-LN. (DOCX 13 kb)

Additional file 5: **Figure S2.** Individual plots of T2*. Plots of T2*mean for individual patients over time. (TIF 46 kb)

Additional file 6: **Figure S3.** Hypoxic subvolume HSV-LN and GTVs on MRI T2*. MRI T2* (ms) showing GTV-T, GTV-LN (red contours) and HSV-LN (green contour) at week 0. (TIF 1376 kb)

## Abbreviations

∆: Delta
BOLD: Blood oxygenation level-dependent
CT: Computed tomography
DCE-MRI: Dynamic contrast enhanced MRI
DWI-MRI: Diffusion weighted MRI
FAZA: ^18^F-fluoroazomycinarabinoside
FDG: ^18^F-fluorodeoxyglucose
FDG-PET: ^18^F-fluorodeoxyglucose PET/CT
FLASH: Multi-echo fast spoiled gradient echo
FMISO: ^18^F-misonidazole
FMISO-PET: ^18^F-misonidazole PET/CT
Gd: Gadolinium
GTV: Gross tumour volume
GTV-LN: Gross tumour volume of metastatic lymph nodes
GTV-T: Gross tumour volume of primary tumour
HNSCC: Squamous cell head-and-neck cancer
HSV: Hypoxic subvolume
HSV-LN: Hypoxic subvolume of lymph node metastasis
HSV-T: Hypoxic subvolume of primary tumour
LN: Lymph nodes
MRI: Magnetic resonance imaging
nonHSV-LN: Non-hypoxic subvolume of lymph node metastasis
nonHSV-T: Non-hypoxic subvolume of primary tumour
NT: Normal tissue
p.i.: Post injection
PET: Positron emission tomography
PET/CT: Positron emission tomography/computed tomography
R2*: Relaxation rate
RCT: Radiochemotherapy
STD: Standard deviation
SUV: Standardized uptake value
SUVmax: Maximum standardized uptake value
SUVmean: Mean standardized uptake value
T: Primary tumour
T1w-MRI: T1-weighted MRI
T2*: Transverse relaxation time
T2w-MRI: T2-weighted MRI
TE: Echo time
TR: Repetition time
