# Correction to: Effect of radiochemotherapy on T2* MRI in HNSCC and its relation to FMISO PET derived hypoxia and FDG PET

**DOI:** 10.1186/s13014-018-1134-7

**Published:** 2018-09-21

**Authors:** Nicole Wiedenmann, Hatice Bunea, Hans C. Rischke, Andrei Bunea, Liette Majerus, Lars Bielak, Alexey Protopopov, Ute Ludwig, Martin Büchert, Christian Stoykow, Nils H. Nicolay, Wolfgang A. Weber, Michael Mix, Philipp T. Meyer, Jürgen Hennig, Michael Bock, Anca L. Grosu

**Affiliations:** 1 Department of Radiation Oncology, Medical Center University of Freiburg, Faculty of Medicine, University of Freiburg, Freiburg, Germany; 2 Department of Radiology, Medical Physics, Medical Center University of Freiburg, Faculty of Medicine, University of Freiburg, Freiburg, Germany; 3 Department of Nuclear Medicine, Medical Center University of Freiburg, Faculty of Medicine, University of Freiburg, Freiburg, Germany; 4 German Cancer Consortium (DKTK), Partner Site Freiburg, Freiburg, Germany; 5 German Cancer Research Center (DKFZ), Heidelberg, Germany; 6 Clinic for Nuclear Medicine, Technische Universität München, Munich, Germany

## Correction

Following the publication of this article [1], the authors noticed that Figs. 2, 3, 4 and 5 were in the incorrect order and thus had incorrect captions.

**Fig. 2:**
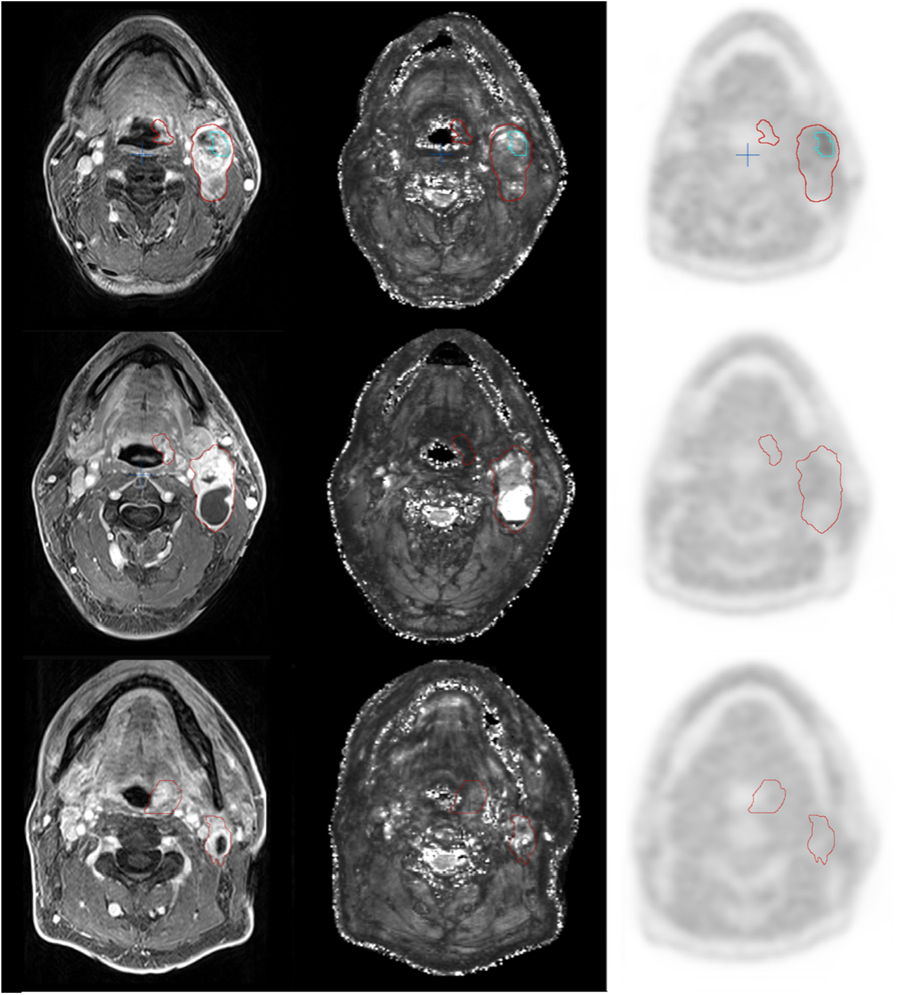
Representative example of imaging modalities MRI T1, T2*, and FMISO-PET. Primary tumour and lymph node metastasis (pt. 5, tonsillar carcinoma) at week 0, 2, and 5 (upper, middle, lower panel): co-registered image sets from MRI T1, MRI T2*, FMISO-PET (left to right). Red contours: GTV-T, GTV-LN. Blue contour: HSV-LN

**Fig. 3:**
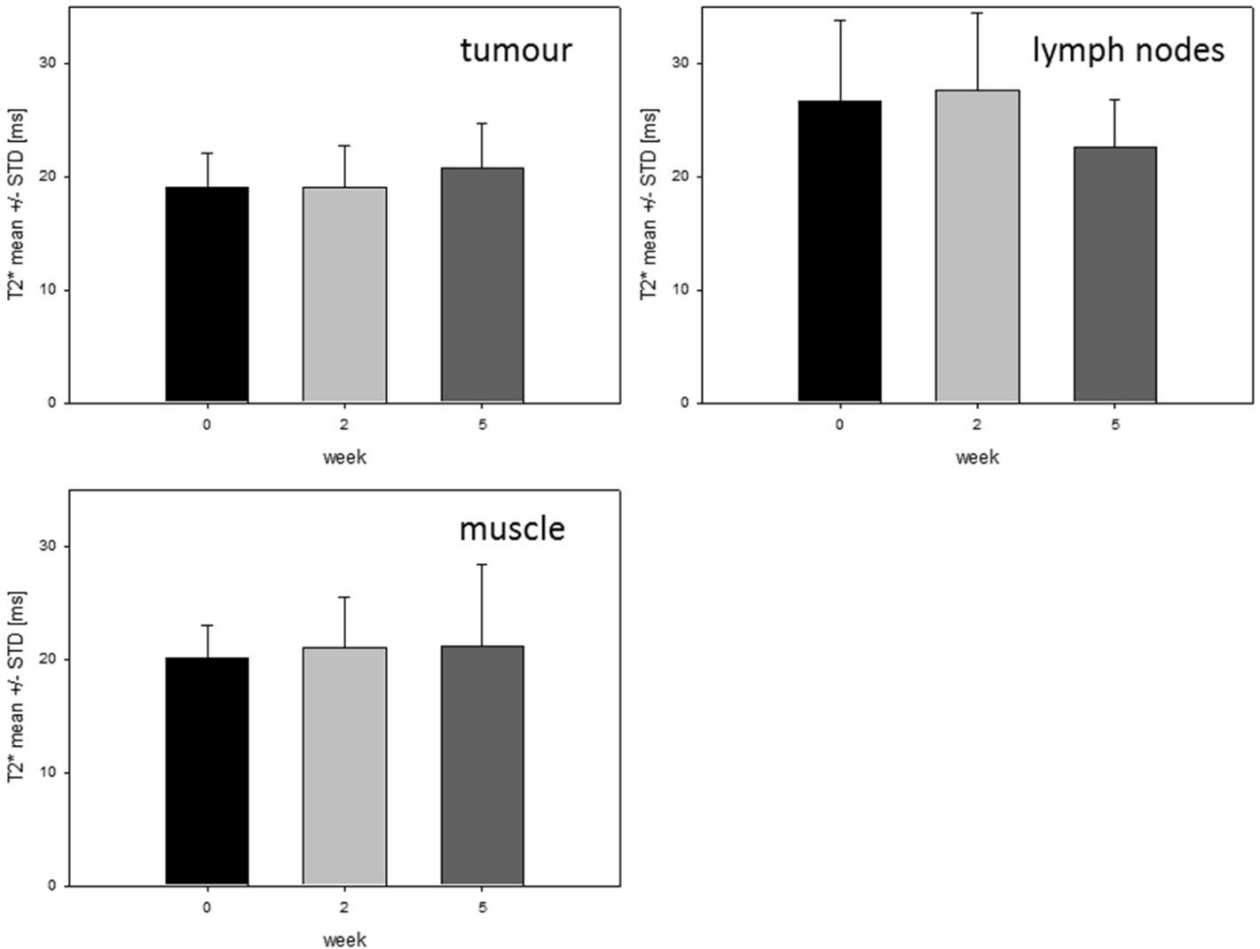
Time course of T2* values within volumes. T2* mean ± STD within tumour, lymph nodes and normal tissue for all patients (*n* = 10)

**Fig. 4:**
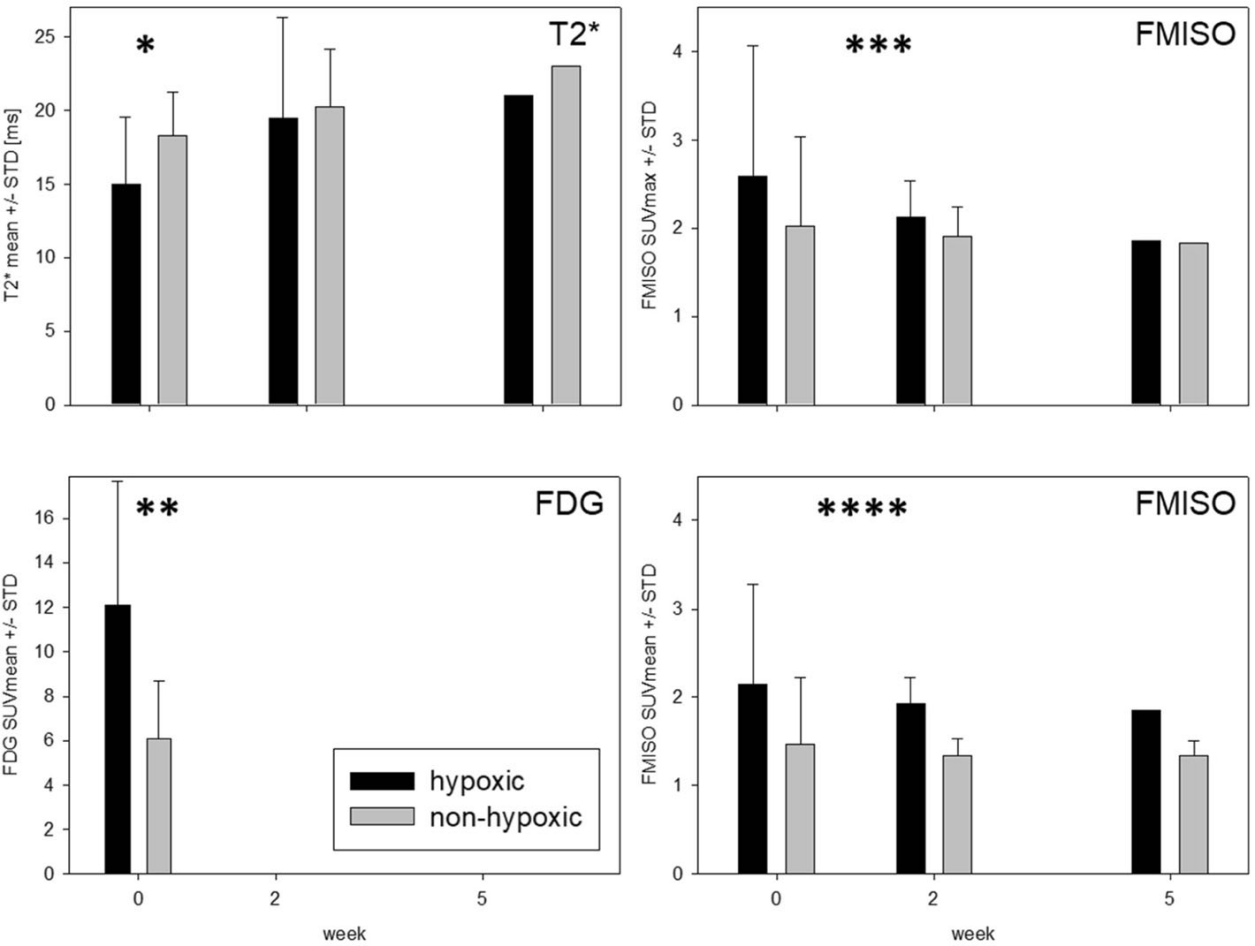
Hypoxic tumour subvolumes: T2* values vs. FDG uptake and FMISO uptake. T2* values (ms) were lower and FDG uptake was higher within hypoxic tumour subvolumes as compared to non-hypoxic tumour subvolumes (*p = 0.051, **p = 0.026). FMISO uptake was higher within hypoxic tumour subvolumes than within non-hypoxic tumour subvolumes (***p = 0.029, *p* = 0.072, ****p = 0.003, *p* = 0.0001)

**Fig. 5:**
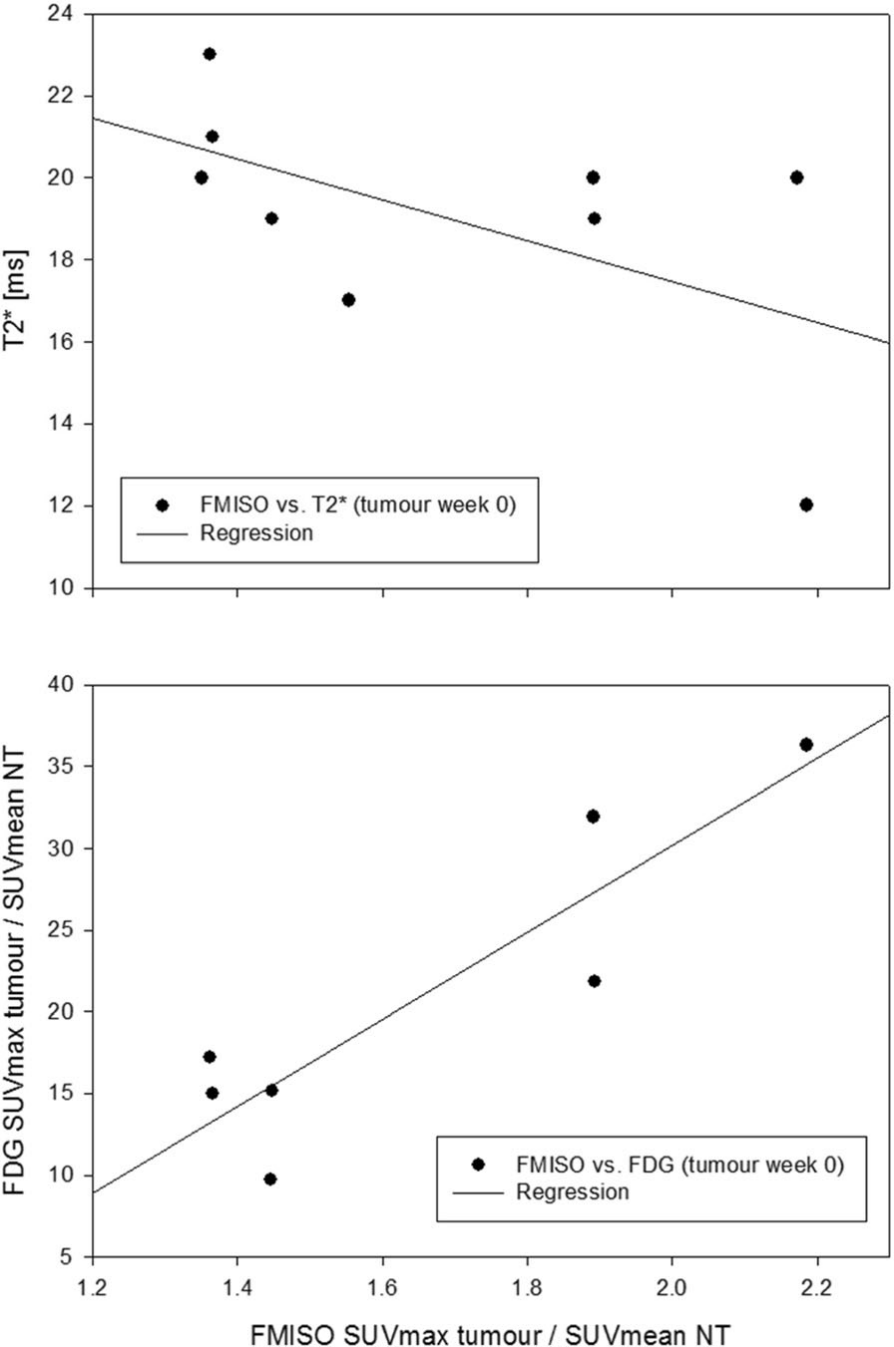
Correlation of FMISO uptake with mean T2* and FDG uptake. Plots showing correlation within GTV-T at baseline

The images that were incorrectly published as Figs. 2, 3, 4 and 5 should have been published as Figs. 4, 5, 2 and 3 respectively.

The correct versions of Figs. 2, 3, 4 and 5 with captions have been included in this Correction.

The original article has been corrected.
