# Supplementary material for: Effect of radiochemotherapy on T2* MRI in HNSCC and its relation to FMISO PET derived hypoxia and FDG PET
Source: Radiat Oncol. 2018 Aug 29;13:159. doi: 10.1186/s13014-018-1103-1 (PMC6114038; doi:10.1186/s13014-018-1103-1)
Supplement: Supplementary file 1 — Table S1. Patient characteristics. (DOCX 15 kb) [file 13014_2018_1103_MOESM1_ESM.docx]

| **Pt** | **Gender** | **Age (yrs.)** | **Tumour site** | **TNM** | **Histology** | **Total/Single Dose** | **Concurrent chemo-therapy** |
| --- | --- | --- | --- | --- | --- | --- | --- |
| 1 | m | 60 | oro-hypopharynx | cT4 cN2c cM0 | PEC, G2, HPV - | 70 / 2 Gy | 2x |
| 2 | m | 53 | oropharynx | cT3 cN2c cM0 | PEC, G3, HPV - | 70 / 2 Gy | 3x |
| 3 | m | 69 | oro-hypopharynx | cT4 cN2c cM0 | PEC, G3, HPV - | 70 / 2 Gy | 1x |
| 4 | m | 54 | hypopharynx | cT4a cN2 cM0 | PEC, G2, HPV - | 70 / 2 Gy | 3x |
| 5 | m | 52 | oropharynx | cT2 cN2b cM0 | PEC, G3, HPV + | 70 / 2 Gy | 3x |
| 6 | m | 61 | hypopharynx | cT3 cN2c cM0 | PEC, G3, HPV - | 70 / 2 Gy | 3x |
| 7 | m | 34 | larynx | cT3 cN2c cM0 | PEC, G2, HPV - | 70 / 2 Gy | 3x |
| 8 | m | 42 | larynx | cT4 cN2b M0 | PEC, G3, HPV - | 70 / 2 Gy | 3x |
| 9 | m | 61 | oro-hypopharynx-larynx | cT4a cN2c M0 | PEC, G2, HPV - | 70 / 2 Gy | 2x |
| 10 | m | 54 | hypopharynx | cT3 cN2c cM0 | PEC, G3, HPV + | 70 / 2 Gy | 3x |

Additional file 1: Table S1

Patient characteristics.
