# Supplementary figures and images for: Effect of radiochemotherapy on T2* MRI in HNSCC and its relation to FMISO PET derived hypoxia and FDG PET
Source: Radiat Oncol. 2018 Aug 29;13:159. doi: 10.1186/s13014-018-1103-1 (PMC6114038; doi:10.1186/s13014-018-1103-1)

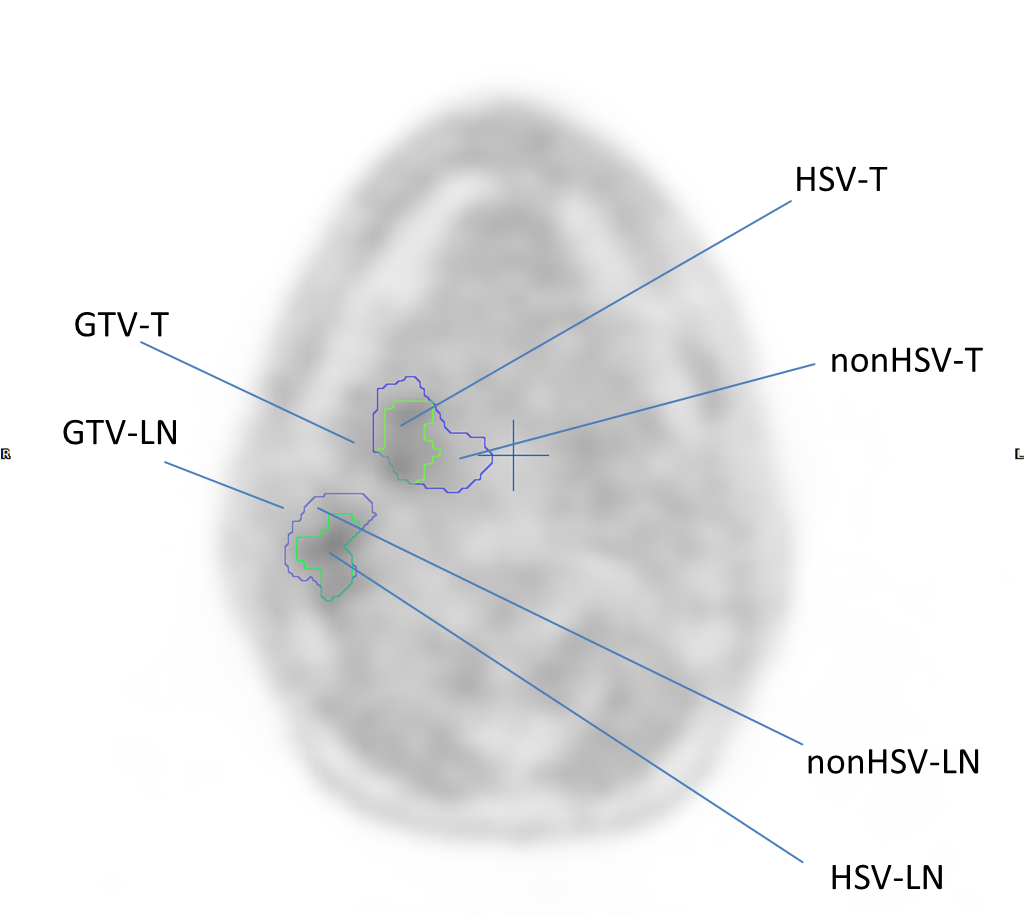

Supplement: Supplementary file 2 — Figure S1. Volumes analysed. FMISO-PET with volumes used for analysis: GTV-T, GTV-LN, HSV-T, nonHSV-T, HSV-LN, nonHSV-LN. (TIF 216 kb) [file 13014_2018_1103_MOESM2_ESM.tif]

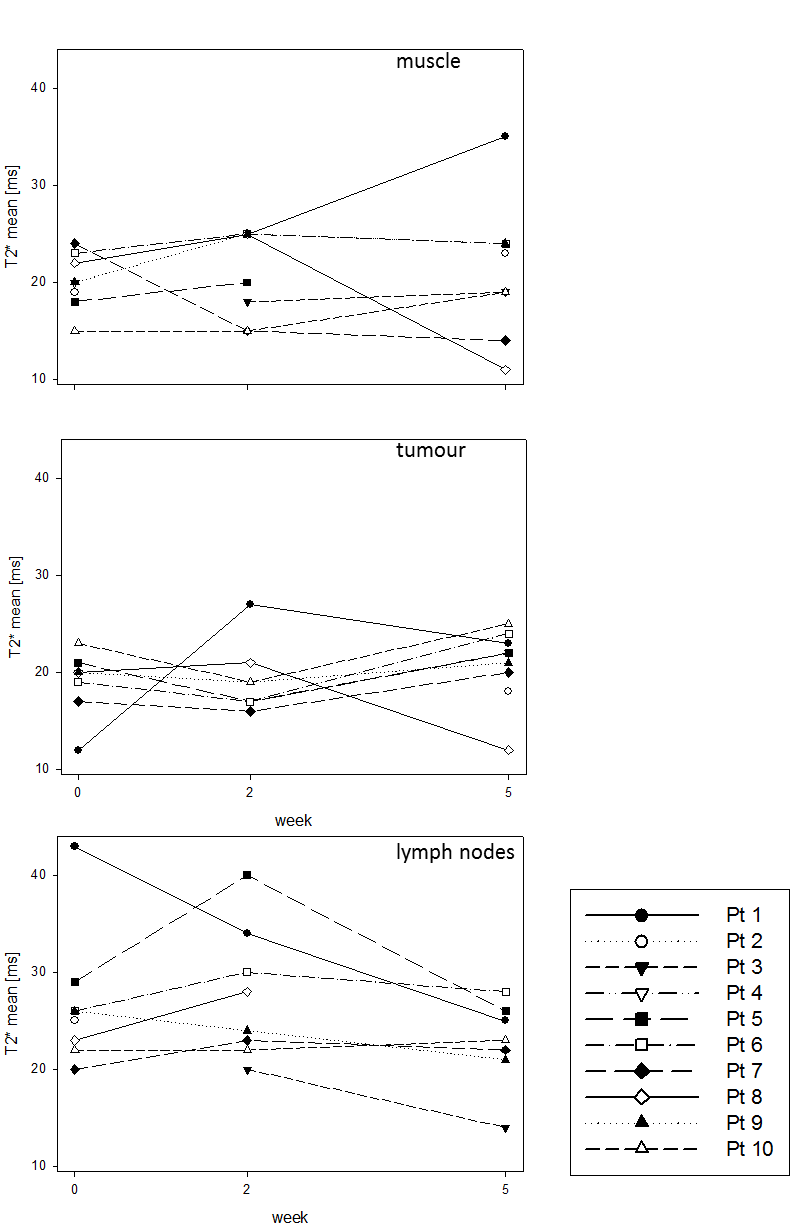

Supplement: Supplementary file 5 — Figure S2. Individual plots of T2*. Plots of T2*mean for individual patients over time. (TIF 46 kb) [file 13014_2018_1103_MOESM5_ESM.tif]

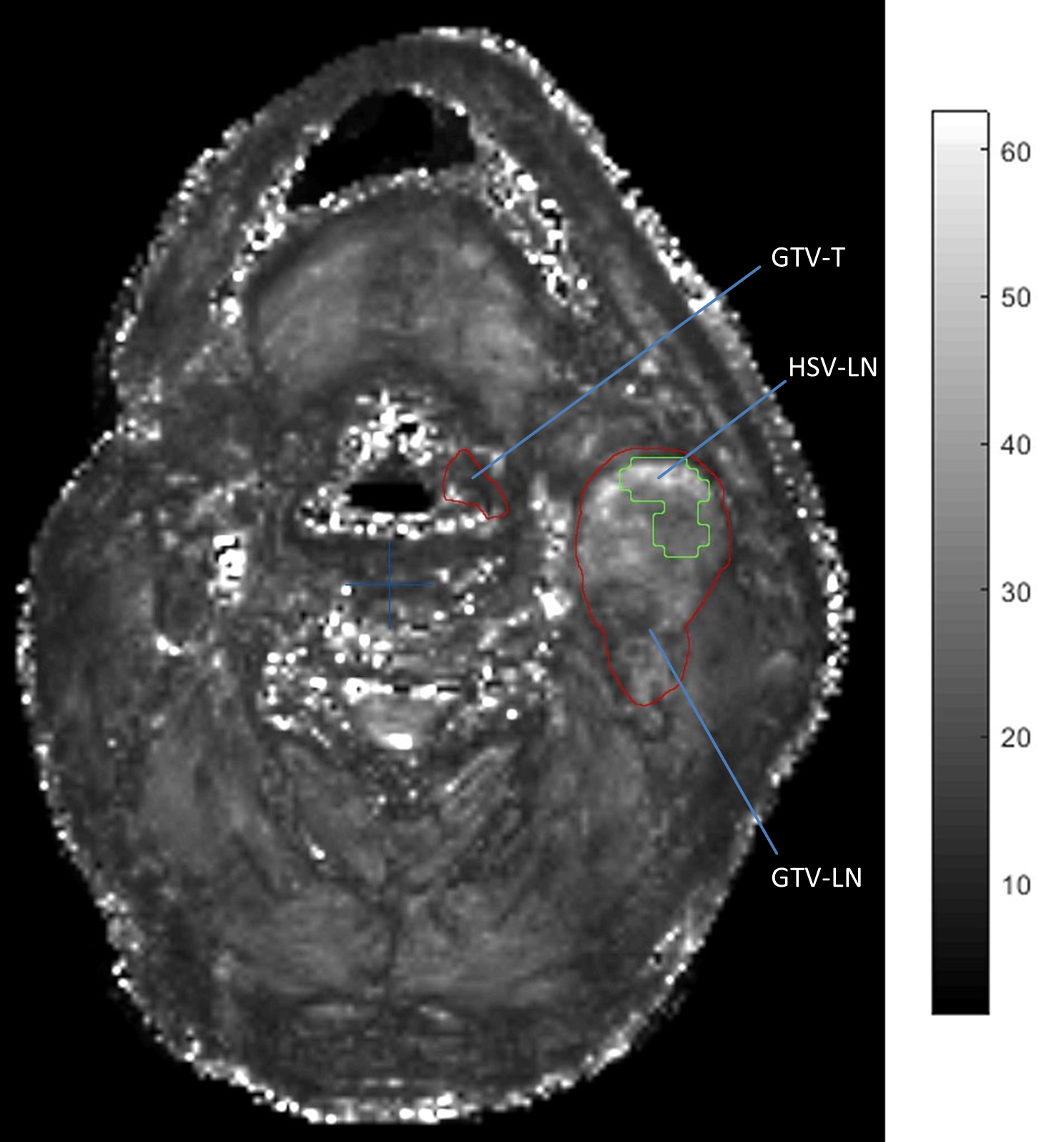

Supplement: Supplementary file 6 — Figure S3. Hypoxic subvolume HSV-LN and GTVs on MRI T2*. MRI T2* (ms) showing GTV-T, GTV-LN (red contours) and HSV-LN (green contour) at week 0. (TIF 1376 kb) [file 13014_2018_1103_MOESM6_ESM.tif]
