# Supplementary material for: Effect of radiochemotherapy on T2* MRI in HNSCC and its relation to FMISO PET derived hypoxia and FDG PET
Source: Radiat Oncol. 2018 Aug 29;13:159. doi: 10.1186/s13014-018-1103-1 (PMC6114038; doi:10.1186/s13014-018-1103-1)
Supplement: Supplementary file 3 — Table S2. T2* values within volumes. Mean, median, and STD for T2*mean (ms) measurements for all patients (n = 10). (DOCX 15 kb) [file 13014_2018_1103_MOESM3_ESM.docx]

| volume week | | mean±STD | | median |
| --- | --- | --- | --- | --- |
|  | | [ms] | | [ms] |
| normal tissue | 0 | | 20.1±2.9 | 22.0 |
|  | 2 | | 21.0±4.6 | 22.5 |
|  | 5 | | 21.1±7.3 | 21.0 |
| GTV-T | 0 | | 19.0±3.1 | 20.0 |
|  | 2 | | 19.1±3.6 | 18.0 |
|  | 5 | | 20.8±3.9 | 22.0 |
| GTV-LN | 0 | | 26.8±7.1 | 25.5 |
|  | 2 | | 27.6±6.8 | 26.0 |
|  | 5 | | 22.6±4.2 | 22.5 |
| HSV-T | 0 | | 15.0±4.6 | 15.0 |
|  | 2 | | 19.5±6.9 | 17.5 |
|  | 5 | | 21.0 | 21.0 |
| nonHSV-T | 0 | | 18.3±2.9 | 19.5 |
|  | 2 | | 20.3±4.0 | 19.0 |
|  | 5 | | 23.0 | 23.0 |
| HSV-LN | 0 | | 28.7±7.6 | 27.5 |
|  | 2 | | 27.2±8.9 | 32.0 |
|  | 5 | | 27.0 | 27.0 |
| nonHSV-LN | 0 | | 28.5±8.5 | 26.0 |
|  | 2 | | 25.6±5.6 | 23.0 |
|  | 5 | | 24.0 | 24.0 |

Additional file 3: Table S2

Mean, median, and STD for T2*mean measurements for all patients (n = 10).
