# Supplementary material for: Effect of radiochemotherapy on T2* MRI in HNSCC and its relation to FMISO PET derived hypoxia and FDG PET
Source: Radiat Oncol. 2018 Aug 29;13:159. doi: 10.1186/s13014-018-1103-1 (PMC6114038; doi:10.1186/s13014-018-1103-1)
Supplement: Supplementary file 4 — Table S3. Number of lymph nodes within GTV-LN. (DOCX 13 kb) [file 13014_2018_1103_MOESM4_ESM.docx]

**Additional file 4: Table S3**

| Patient # | Lymph nodes present in the GTV-LN |
| --- | --- |
| 1 | 2 |
| 2 | 3 |
| 3 | 2 |
| 4 | 2 |
| 5 | 1 |
| 6 | 5 |
| 7 | 4 |
| 8 | 1 |
| 9 | 10 |
| 10 | 9 |
